# Pattern of uveitis in a referral ophthalmology center in Northeastern Thailand

**DOI:** 10.1186/s12348-024-00400-6

**Published:** 2024-05-31

**Authors:** Trakanta Wannapanich, Waraporn Chuenchaem, Patanaree Luanratanakorn, Wipada Laovirojjanakul

**Affiliations:** Department of Ophthalmology, Faculty of Medicine, Khon Kaen University, Khon Kaen, Thailand

## Abstract

**Purpose:**

To report the characteristics and epidemiology of uveitis in a university-based referral center in northeastern Thailand and review the uveitis patterns present in various regions worldwide.

**Methods:**

A retrospective review of all medical records for new patients visiting the uveitis clinic at Srinagarind hospital, Khon Kaen University, between August 2016 and June 2021, was conducted.

**Results:**

A total of 522 uveitis patients were included in this study. Disease etiologies were categorized as non-infectious 35.8% (187/522), infectious 32.8% (171/522), and undetermined cause 31.4% (164/522). Specific diagnoses were established in 68.6% of cases. Vogt-Koyanagi-Harada (VKH) (14.2%) was identified as the most common specific diagnosis, and tuberculosis (6.7%) ranked highest among infectious causes.

**Conclusions:**

Although non-infectious uveitis is observed predominantly in this region, the proportion of infectious uveitis is relatively more common than in developed countries. We have found no cause for one-third of our patients despite the utilization of PCR and serology for diagnostic purposes.

## Introduction

Uveitis comprises a group of ocular disorders characterized by inflammation within the eye, leading to a significant visual impairment, accounting for 5–10% of causes of legal blindness [1, 2]. The etiology of uveitis varies globally due to factors such as geographic location and demographic differences among populations. Epidemiological studies on uveitis in Thailand have, to date, primarily focused on the central, northern and southern region [3–8]. This study aims to report the patterns of uveitis at the Department of Ophthalmology, Srinagarind Hospital, a referral center for ophthalmology in the northeastern part of Thailand.

## Materials and methods

A retrospective descriptive cross-sectional study was conducted on all uveitis patients visiting the Department of Ophthalmology, Srinagarind Hospital, from August 2016 and June 2021. The study adhered to the principles of the Declaration of Helsinki and received approval from the Ethics Committee of Khon Kaen University (HE611037). Inclusion criteria covered patients diagnosed with all types of uveitis. The exclusion criteria were all types of endophthalmitis (including post-operative, post-traumatic, and endogenous), episcleritis, scleritis, and peripheral ulcerative keratitis. Patient data, including age, gender, geographic location, laterality, anatomical involvement, type of uveitis, etiology, disease course, disease activity, medical and surgical treatment, visual prognosis, and complications, were extracted from medical records. Patients were anatomically classified based on the Standardization of Uveitis Nomenclature (SUN) criteria [9]. A comprehensive eye examination involving slit-lamp biomicroscopy, applanation tonometry, and dilated fundus examination, was conducted for all patients. Additional investigations, such as fundus fluorescein angiography, indocyanine green angiography, and optical coherence tomography, were determined by a uveitis specialist. Specific diagnoses were confirmed or strongly suspected based on clinical history, ocular findings, laboratory results, and ancillary tests, with definite diagnoses relying on the best available evidence during the study period.

Tests for infectious causes included Venereal Disease Research Laboratory (VDRL), *Treponema pallidum* hemagglutination (TPHA) test, interferon gamma releasing assay (IGRA; QuantiFERON-TB Gold test), polymerase chain reaction (PCR) for *Mycobacterium tuberculosis*, toxoplasma antibody testing, toxocara antibody testing, serum enzyme-linked immunosorbent assay (ELISA) for specific parasites (*Gnathostoma* spp., *Angiostrongylus* spp.), human immunodeficiency virus (HIV) antibody test, viral antibody testing, PCR for herpes viruses, microbiological cultures, and any evidence of ongoing systemic infection at the time of uveitis onset. The diagnosis of ocular toxocariasis primarily relied on clinical findings such as retinal granuloma, tractional retinal detachment, vitreous haze and, when available, correlation with serological tests. The diagnosis of ocular parasitic infections also included the identification of parasites in any part of the eye, such as *Gnathostoma* spp., *Angiostrongylus* spp., and cysticercosis. Investigations for non-infectious etiologies encompassed anti-nuclear activity (ANA), rheumatoid factor, anti-ds-DNA, anti-neutrophil cytoplasmic antibodies (ANCA), and human leukocyte antigen typing (HLA-B27). When necessary, we performed anterior chamber paracentesis to obtain aqueous fluid specimens. Additionally, vitreous samples were collected through diagnostic pars plana vitrectomy. The choice of ancillary tests was determined case-by-case based on clinical suspicions.

To assess visual impairment, low vision was defined as best corrected visual acuity (BCVA) less than 6/18 using Snellen chart but equal to or better than 3/60 in the better eye. Blindness was defined as BCVA less than 3/60 in the better eye [10]. Complication assessment included the identification of glaucoma when intraocular pressure (IOP) exceeding 21 mmHg with further evidences of glaucomatous optic neuropathy. Ocular hypertension was defined as a transient IOP elevation greater than 21 mmHg without the need for IOP-lowering drugs. Ocular hypotony was characterized by an IOP lower than 6 mmHg. Secondary cataract was considered when patients developed nuclear sclerosis and/or posterior subcapsular cataracts to a greater degree than expected for their age or exhibited asymmetrical cataracts with greater severity in the affected eye for unilateral uveitis patients. Data recording and descriptive statistical analysis were performed using Microsoft Excel (version 16.16.4, Microsoft, Raymond, WA).

## Results

A total of 522 patients from the uveitis clinic were included in this study, with 51.5% (269/522) being male and 48.5% (253/522) female. The average age was 44. Unilateral diseases accounted for 48.1% (251/522) while 51.9% (271/522) of patients had uveitis bilaterally.

Anatomical classification of the 522 patients revealed that panuveitis was the most common presentation, observed in 52.3% (273/522), followed by anterior uveitis and posterior uveitis at 24.1% (126/522) and 19.0% (99/522), respectively (Table 1).

**Table 1:** Demographic and clinical characteristics by anatomical involvement

Characteristics	Total (*n* = 372)		Anterior (*n* = 82)		Anterior and intermediate (*n* = 5)		Intermediate (*n* = 9)		Posterior (*n* = 70)		Panuveitis (*n* = 206)	
	*n*	%	*n*	%	*n*	%	*n*	%	*n*	%	*n*	%
**Age**												
Mean ± SD (years)	43.7 ± 15.9		45.8 ± 17.3		46.9 ± 11.7		44.2 ± 20.2		42.3 ± 15.7		43.2 ± 15.1	
≤16	26	5.0	7	5.6	0	0.0	2	12.5	5	5.1	12	4.4
17–59	406	77.8	87	69.0	7	87.5	10	62.5	78	78.8	224	82.1
≥60	90	17.2	32	25.4	1	12.5	4	25.0	16	16.2	37	13.6
**Gender**												
Male	269	51.5	55	43.7	5	62.5	6	37.5	50	50.5	153	56.0
Female	253	48.5	71	56.3	3	37.5	10	62.5	49	49.5	120	44.0
**Laterality**												
Unilateral	251	48.1	78	61.9	2	25.0	7	43.8	62	62.6	101	37.0
Bilateral	271	51.9	48	38.1	6	75.0	9	56.3	37	37.4	172	63.0

The diagnoses of all patients are detailed in Table 2. Disease etiologies were categorized as non-infectious 35.8% (187/522), infectious 32.8% (171/522), and undetermined cause 31.4% (164/522). Vogt-Koyanagi-Harada (14.2%) was the most frequent cause of uveitis among the non-infectious group, followed by Behcet’s disease (9.0%) and HLA-B27 associated anterior uveitis (4.2%). There were three cases diagnosed with intraocular lymphoma, with two cases presenting with unilateral posterior uveitis and one case presenting with bilateral panuveitis. Coat’s disease was included in the uveitis clinic because the patient was consulted due to retinal infiltration suspected as posterior uveitis. Therefore, Coat’s disease may be categorized under the pediatric masqueraders group. Among the infectious group, ocular tuberculosis (6.7%), herpetic anterior uveitis (5.2%) and ocular toxoplasmosis (5.0%) were the most common pathogens (Table 2).

**Table 2:** Patient characteristics listed by anatomical site and definite diagnosis

Etiology	Total (*n* = 522)		Anterior (*n* = 126)		Anterior and intermediate (*n* = 8)		Intermediate (*n* = 16)		Posterior (*n* = 99)		Panuveitis (*n* = 273)	
	*n*	%	*n*	%	*n*	%	*n*	%	*n*	%	*n*	%
**Non-Infectious**												
VKH	74	14.2	-	-	-	-	-	-	6	6.1	68	24.9
Behcet’s disease	47	9.0	-	-	-	-	-	-	6	6.1	41	15.0
HLA-B27 AU	22	4.2	20	15.9	2	25.0	-	-	-	-	-	-
Sarcoidosis	8	1.5	-	-	-	-	-	-	-	-	8	2.9
MCP	6	1.1	-	-	-	-	-	-	1	1.0	5	1.8
AS	5	1.0	5	4.0	-	-	-	-	-	-	-	-
Lymphoma	3	0.6	-	-	-	-	-	-	2	2.0	1	0.4
Vasoproliferative tumor	3	0.6	-	-	-	-	-	-	2	2.0	1	0.4
SO	3	0.6	-	-	-	-	-	-	-	-	3	1.1
SLE	2	0.4	1	0.8	-	-	-	-	-	-	1	0.4
SC	2	0.4	-	-	-	-	-	-	2	2.0	-	-
FHI	1	0.2	1	0.8	-	-	-	-	-	-	-	-
PAN	1	0.2	1	0.8	-	-	-	-	-	-	-	-
RA	1	0.2	1	0.8	-	-	-	-	-	-	-	-
GPA	1	0.2	1	0.8	-	-	-	-	-	-	-	-
JIA	1	0.2	1	0.8	-	-	-	-	-	-	-	-
Ampiginous choroiditis	1	0.2	-	-	-	-	-	-	1	1.0	-	-
AZOOR	1	0.2	-	-	-	-	-	-	1	1.0	-	-
Coats disease	1	0.2	-	-	-	-	-	-	1	1.0	-	-
Leukemia	1	0.2	-	-	-	-	-	-	-	-	1	0.4
Phacoantigenic uveitis	1	0.2	-	-	-	-	-	-	-	-	1	0.4
RP	1	0.2	1	0.8	-	-	-	-	-	-	-	-
Brimonidine-induced	1	0.2	1	0.8	-	-	-	-	-	-	-	-
**Total**	187	35.8	33	0.3	2	25.0	-	-	22	0.2	130	47.6
**Infectious**												
TB	35	6.7	3	2.4	-	-	-	-	6	6.1	26	9.5
Herpetic AU	27	5.2	27	21.4	-	-	-	-	-	-	-	-
Toxoplasmosis	26	5.0	-	-	-	-	-	-	16	16.2	10	3.7
ARN/PORN	22	4.2	-	-	-	-	-	-	6	6.1	16	5.9
CMV	19	3.6	7	5.6	-	-	-	-	2	2.0	10	3.7
Syphilis	13	2.5	2	1.6	-	-	-	-	2	2.0	9	3.3
Toxocariasis	13	2.5	-	-	-	-	-	-	6	6.1	7	2.6
Neuroretinitis	9	1.7	-	-	-	-	-	-	9	9.1	-	-
Rubella	2	0.4	-	-	-	-	-	-	-	-	2	0.7
Gnathostomiasis	1	0.2	-	-	1	12.5	-	-	-	-	-	-
Cysticercosis	1	0.2	-	-	-	-	-	-	-	-	1	0.4
Angiostrongy-liasis	1	0.2	-	-	-	-	-	-	-	-	1	0.4
DUSN	1	0.2	-	-	-	-	-	-	-	-	1	0.4
Post-murine typhus uveitis	1	0.2	-	-	-	-	-	-	-	-	1	0.4
**Total**	171	32.8	39	31.0	1	12.5	-	-	47	47.5	84	30.8
Undetermined	164	31.4	54	42.9	5	62.5	16	100.0	30	30.3	59	21.6

AU, anterior uveitis; ARN, acute retinal necrosis; AS, ankylosing spondylitis; AZOOR, acute zonal occult outer retinopathy; CMV, cytomegalovirus; DUSN, diffuse unilateral subacute neuroretinitis; FHI, Fuchs heterochromic iridocyclitis; GPA, granulomatosis with polyangiitis; herpetic AU, herpetic anterior uveitis; HLA-B27 AU, HLA-B27 associated anterior uveitis; HZV, herpes zoster virus; M, male; PORN, progressive outer retinal necrosis; RA, rheumatoid arthritis; SC, serpiginous choroiditis; SO, sympathetic ophthalmia; TB, *Mycobacterium tuberculosis*; VKH, Vogt-Koyanagi-Harada disease

In this study, 21 patients had human immunodeficiency virus (HIV) infection, with 17 of them associated with infectious conditions: CMV retinitis (7 cases), ocular syphilis (6 cases), herpetic retinitis (2 cases), ocular tuberculosis (1 case), and ocular toxoplasmosis (1 case).

Immunomodulatory agents were prescribed for 138 patients (26.4%), including methotrexate, azathioprine, cyclosporine A, and mycophenolate mofetil. Most patients requiring these drugs, either as monotherapy or combination therapy, were mostly diagnosed with VKH (45 patients) and Behcet’s disease (40 patients).

### Visual assessment

Out of 271 patients with bilateral uveitis, blindness occurred in 27 patients (10.0%), mostly caused by VKH (9 patients). Low vision was recorded in 69 patients (25.5%), with Behcet’s disease (12 patients) being the leading cause, followed by VKH (9 patients). Unilateral uveitis led to blindness in the affected eye in 62 out of 251 patients (24.7%) with the majority caused by acute retinal necrosis (9 patients) and herpetic anterior uveitis (7 patients).

### Complications

The most frequent ophthalmic complication found in this study was glaucoma, occurring in 20.9% of patients, followed by retinal detachment (6.3%) and cystoid macular edema (5.6%). Other complications included cataracts, ocular hypertension, epimacular membrane, band keratopathy, corneal decompensation, ocular hypotony, and phthisis bulbi. (Table 3)

**Table 3:** Complications of uveitis found in this study

Complications	No. of patients (%)
Glaucoma Retinal detachment Cystoid macular edema Cataract Ocular hypertension Epimacular membrane Band keratopathy Corneal decompensation Hypotony Phthisis bulbi	109 (20.9%) 33 (6.3%) 29 (5.6%) 26 (5.0%) 20 (3.8%) 11 (2.1%) 8 (1.5%) 7 (1.3%) 4 (0.8%) 2 (0.4%)

## Discussion

This study delineates the pattern of uveitis among patients at a tertiary eye center in Northeast Thailand. Tables 4 and 5 present a comparative analysis of findings between this study and others conducted in Thailand and globally. The results indicate a slight male predominance, aligning with previous reports that demonstrated comparable numbers between genders [3–8, 11–22]. The mean age of patients was 44 years, with the majority falling within the middle age range (17–60 years old), consistent with previous studies [3–8, 12–19].

**Table 4:** Pattern of uveitis in Thailand

Publication	Pathanapitoon [3]	Sittivarakul [5]	Silpa-archa [6]	Sukavatcharin [7]	Keorochana [8]	This study
Year	2008	2013	2015	2016	2020	2024
Region	Northern	Southern	Central	Central	Central	Northeastern
Sample size	200	254	446	758	586	522
Mean age (Years)	38.4	42.6	42.1	45.6	46.3	43.7
Gender M: F	1:0.9	1:0.8	1:1.2	1:1.1	1:0.8	1:0.9
**Anatomical location (%)**						
Most common	Posterior 46.0%	Anterior 35.4%	Anterior 44.8%	Anterior 46.3%	Anterior 50.0%	Panuveitis 52.3%
	Anterior 24.5%	Panuveitis 34.6%	Panuveitis 40.0%	Panuveitis 34.7%	Panuveitis 25.6%	Anterior 24.1%
	Panuveitis 22.0%	Posterior 19.7%	Posterior 14.3%	Posterior 14.6%	Posterior 12.3%	Posterior 19.0%
**Etiology (%)**						
Infection	44.0%	27.1%	13.3%	42.5%	20.0%	32.8%
Non-infection	43.0%	43.8%	38.3%	41.0%	44.0%	35.8%
Undetermined	13.0%	29.1%	48.4%	16.5%	36.0%	31.4%
**Specific diagnosis (%)**						
Most common	CMV 27%	VKH 11.0%	VKH 22.4%	Herpetic AU 17.2%	Behcet’s 15.4%	VKH 14.2%
	VKH 11%	HLA-B27 AU 7.9%	Behcet’s 6.7%	VKH 13.5%	HLA-B27 AU 12.8%	Behcet’s 9.0%
	Toxoplasmosis 6%	Toxoplasmosis 7.1%	Herpetic AU 5.8%	CMV 12.7%	VKH 6.8%	TB 6.7%
**Infectious pathogen (%)**						
Most common	CMV 27%	Toxoplasmosis 7.1%	Herpetic AU 5.8%	Herpetic AU 17.2%	CMV 6.2%	TB 6.7%
	Toxoplasmosis 6%	Herpetic AU 4.7%	TB 2.2%	CMV 12.7%	TB 4.9%	Herpetic AU 5.2%
	HSV/VZV 3.5%	ARN 3.9%	Toxoplasmosis 1.6%	TB 8.6%	HSV/VZV 2.9%	Toxoplasmosis 5.0%

AU, anterior uveitis; ARN, acute retinal necrosis; CMV, cytomegalovirus; F, female; Herpetic AU, herpetic anterior uveitis; HLA-B27 AU, HLA-B27 associated anterior uveitis; HSV, herpes simplex virus; M, male; TB, *Mycobacterium tuberculosis*; VKH, Vogt-Koyanagi-Harada disease; VZV, varicella zoster virus

**Table 5:** Pattern of uveitis in different countries from recent studies

Study	Country	Year	Sample size	Mean age (Yrs)	Gender M: F	Location (%)				Etiology (%)			Three most common causes	Three most common infectious pathogens
						Ant-	Int-	Post-	Pan-	Infect-	Non-inf-	Idio-		
This study	Thailand	2024	522	43.7	1:0.9	24.1	4.6*	19.0	52.3	32.8	35.8	31.4	VKH, Behcet’s, TB	TB, Herpetic AU, Toxoplasmosis
Alarfaj [34]	Saudi Arabia	2023	222	38.5	1:1.2	51.1	12.8	10.0	26.0	11.3	45.0	43.7	VKH, Behcet’s, FHI, JIA	TB, Herpetic uveitis, Toxoplasmosis
Alawneh [35]	Jordan	2023	221	36.0	1:1	57.5	12.7	5.9	15.4	4.5	49.8	45.7	Behcet’s, SNSA, JIA	Herpetic AU, Toxoplasmosis, TB
Kalogeropoulos [36]	Greece	2023	6191	40.6	1:1.13	59.1	6.0	21.9	13.1	31.1	33.9	32.8	Herpetic uveitis, Toxoplasmosis, Sarcoidosis	Herpetic uveitis, Toxoplasmosis, TB
Solomon [27]	Ethiopia	2022	82	33.8	1:0.5	57.0	20.7	9.8	12.2	18.3	9.8	72.0	Herpetic uveitis, Toxoplasmosis, CMV retinitis	Herpetic uveitis, Toxoplasmosis, CMV retinitis
Rajan [25]	Malaysia	2022	1199	NA	1:0.9	46.7	11.3	20.8	21.2	34.3	8.5	57.2	TB, Viral uveitis, Toxoplasmosis	TB, Viral uveitis, Toxoplasmosis
Pandurangan [26]	India	2022	102	39.1	1:0.6	23.4	11.3	46.8	18.5	23.4	19.9	56.7	TB, Sympathetic ophthalmitis, HLA-B27	TB, Toxoplasmosis, Herpetic uveitis
Suzuki [37]	Japan	2021	732	56.4	1:1.2	33.1	1.5	7.1	58.3	NA	NA	38.7	Sarcoidosis, herpetic iridocyclitis, intraocular lymphoma	Herpetic iridocyclitis, Bacterial endoph, CMV retinitis
Sonoda [38]	Japan	2021	5378	NA	1:1.3	36	2.5	14	44.9	15.4	47.2	36.6	Sarcoidosis, VKH, herpetic iritis	Herpetic iritis, ARN, CMV retinitis
Hermann [39]	Portugal	2021	545	47.9	1:0.8	47.5	5.5	26.4	4.2	18.0	49.9	32.1	Axial spondyloarthritis, Behcet’s, sarcoidosis	TB, Herpetic uveitis, Toxoplasmosis
Hao [40]	China	2021	2000	39.9	1:1.1	39.3	5.5	6.0	49.3	NA	NA	45.2	NA	NA
García-Aparicio [41]	Spain	2021	389	47.0	1:1.4	83.2	4.3	3.2	9.8	13.4	38.6	48.0	NA	Herpes, CMV, Toxoplasmosis
Abdelwareth [42]	Egypt	2021	313	30.0	1:0.5	33.2	0.6	12.8	53.4	23.4	52.0	24.6	Behcet’s, Parasitic uveitis, VKH	Parasites, Herpetic uveitis, Endoph
Tolesa [43]	Ethiopia	2020	98	NA	1:0.5	74.5	1.0	9.2	15.3	15.3	7.1	77.6	Herpetic uveitis, Toxoplasmosis, FHI	Herpetic uveitis, Toxoplasmosis, Toxocariasis = ARN = Syphilis
Bro [30]	Sweden	2020	2483	NA	NA	93	1	5	1	NA	NA	86.1	Herpetic uveitis, IBD, RA	Herpetic uveitis, Toxoplasmosis, HIV
Borde [44]	India	2020	210	46.6	1:1	47.1	31.9	12.9	8.1	25.7	26.2	48.1	TB, Herpetic AU, Spondyloarthropathy (SNSA + HLA-B27)	TB, Herpetic uveitis, Toxoplasmosis
Hart [45]	Australia	2019	1236	46.3	1:0.9	74.4	5.8	15.2	4.5	13.4	26.4	60.2	HLA-B27, Herpetic AU, AS	HSV, VZV, Toxoplasmosis
Gray [46]	Northern Ireland	2019	255	NA	NA	0.6	19.4	80.0**		NA	NA	NA	Sarcoidosis, Toxoplasmosis, ARN	Toxoplasmosis, ARN, TB
El Latif [19]	Egypt	2019	1315	33.5	1:0.9	36.4	23.7	23.0	16.9	31.1	38.8	30.1	TB, Sarcoidosis, Behcet’s	TB, Toxoplasmosis, Toxocariasis
Shirahama [47]	Japan	2018	750	56.4	1:1.1	38.5	1.6	12.5	47.3	20.4	38.9	40.7	Herpetic iridocycliis, Sarcoidosis, Behcet’s	Herpetic uveitis, Bacterial endoph, Fungal endoph
Rahman [18]	Bangladesh	2018	652	32.3	1:0.9	39.3	22.2	22.1	16.4	20.2	33.1	46.6	TB, HLA-B27, VKH	TB, Toxoplasmosis, Viral AU
Hosseini [48]	Iran	2018	235	35.8	1:1.5	37.0	11.9	4.3	46.8	19.6	51.9	28.5	Behcet’s, VKH, Herpetic uveitis	Herpetic uveitis, TB, Toxoplasmosis
Biswas [28]	India	2018	352	NA	1:0.8	35.2	30.1	25.0	9.7	NA	NA	33.8	TB, HLA-B27, VKH	TB, Toxoplasmosis, ARN
Win [20]	Myanmar	2017	139	36.3	1:0.9	45.3	10.1	23.7	20.9	54.7	10.8	34.5	TB, HIV, Endogenous endoph	TB, HIV, CMV
Siak [49]	Sri Lanka	2017	750	31.3	1:0.5	38.0	20.0	24.9	17.1	16.7	18.7	64.7	TB, Toxoplasmosis, SNSA	TB, Toxoplasmosis, Herpetic AU
Rautenbach [50]	South Africa	2017	198	38.0	1:1.1	40.9	2.5	19.2	37.4	47.0	18.2	34.8	Syphilis, HLA-B27, Toxoplasmosis	Syphilis, Toxoplasmosis, TB
Luca [17]	Italy	2017	990	44***	1:1.4	53.5	7.5	16.2	22.8	30.4	46.3	23.3	Herpetic AU, FHI, HLA-B27	Herpetic AU, TB, Toxoplasmosis
Abaño [51]	Philippines	2017	595	38.5	1:1.2	49.6	6.9	20.2	23.4	25.2	20.5	54.3	TB, VKH, Toxoplasmosis	TB, Toxoplasmosis, Toxocariasis
Dogra [52]	India	2017	1912	36.6	1:08	43.0	10.7	24.6	16.2	33.4	27.1	39.4	HLA-B27, FHI, JIA	Herpetic uveitis, TB, Syphilis
Nguyen [23]	Vietnam	2017	212	40.5	1:0.8	46.2	14.2	22.2	17.5	27.4	36.3	36.3	VKH, TB, Behcet’s	TB, Herpetic AU, Toxoplasmosis
Siak [53]	Singapore	2016	1249	45.8	1:1	64.4	7.4	10.2	18.1	30.9	69.1	25.0	HLA-B27, CMV AU, VKH	CMV, Herpetic AU, TB
Gao [54]	China	2016	606	33.8	1:1.1	26.6	0.8	7.3	65.3	6.3	54.7	39.0	VKH, Behcet’s, HLA-B27	TB, ARN, Syphilis
Jones [55]	United Kingdom	2015	3000	NA	1:1.2	46.0	11.1	21.8	21.1	NA	NA	31.2	FHI, Sarcoidosis, Toxoplasmosis	Toxoplasmosis, TB, VZV
Al-Shakarchi [16]	Iraq	2014	318	36.2	NA	24.5	6.3	38.7	30.5	28.9	37.1	34.0	Toxoplasmosis, VKH, TB	Toxoplasmosis, TB, Herpetic AU
Acharya [15]	Unitied States	2013	224	NA	1:1.3	72.3	6.3*	21.4**		NA	NA	NA	Herpetic uveitis, Histoplasmosis, Toxoplasmosis = PSS	Herpetic uveitis, Histoplasmosis, Toxoplasmosis

Ant-, anterior; AU, anterior uveitis; ARN, acute retinal necrosis; CMV, cytomegalovirus; Endoph, endophthalmitis; F, female; FHI, Fuchs heterochromic iridocyclitis; Herpetic AU, herpetic anterior uveitis; HLA-B27 AU, HLA-B27 associated anterior uveitis; HSV, herpes simplex virus; IBD, inflammatory bowel disease; Idio-, idiopathic; Infect-, infectious; Int-, intermediate; M, male; NA, not available; Non-inf-, non-infectious; Pan-, panuveitis; Post-, posterior; SNSA, seronegative spondyloarthropathy; TB, *Mycobacterium tuberculosis*; RA, rheumatoid arthritis; VKH, Vogt-Koyanagi-Harada disease; VZV, varicella zoster virus

* Patients with anterior and intermediate uveitis were classified as intermediate

** Posterior uveitis and panuveitis cases were analyzed together

*** Median

Panuveitis emerged as the most prevalent anatomical location of uveitis in this study (52.3%). In contrast, anterior uveitis was the common anatomical location predominantly found in previous studies [4–8, 12, 13, 15, 17–20]. These findings indicate VKH as the most common specific etiology overall. This correlates with several studies from Thailand [5, 6], Japan [14], and Vietnam [23]. Notably, the number of patients with non-infectious entities exceeded those with infectious uveitis. Interestingly, the percentage of infectious uveitis was relatively high compared to reports from other countries [11–19].

Intraocular tuberculosis (IOTB), diagnosed based on the classification mentioned earlier [24], accounted for the largest number of patients in the infectious group, frequently manifesting with chorioretinitis (13 patients) and retinal vasculitis (12 patients). The finding aligns with studies by Sukavatcharin et al [7], Rajan et al [25], and Nguyen et al [23], which reported tuberculosis diagnoses in 8.6%, 8.7, and 9.0% of uveitis patients, respectively. Additional studies has highlighted tuberculosis as the most prevalent infectious pathogens, constituting 32% in Myanmar [20] and 18.5% in India [26]. Conversely, studies in other regions of Thailand reported herpetic uveitis, toxoplasmosis, or CMV uveitis as the most prevalent infectious etiologies [3–8]. In anterior uveitis patients in this study, viral infection emerged as the most common cause of inflammation, consistent with studies from central Thailand [6], Vietnam [23], Malaysia [25], and Ethiopia [27], rather than HLA-B27 or ankylosing spondylitis, which were frequently more reported in most Asian countries [3, 5, 7, 18, 21, 26, 28], except Myanmar which reported tuberculosis [20]. Toxoplasmosis was a common specific diagnosis in the posterior uveitis group, similar to reports from India [29], North Africa [12], Iraq [16], and Ethiopia [27].

Risk factors associated with infectious uveitis cases depend on the infectious etiologies involved. Given that Thailand is an endemic area for tuberculosis infection, it is considered one of the risk factors for IOTB, along with documented exposure to tuberculosis and clinical evidence of extraocular tuberculosis manifestations. Additionally, most patients with CMV retinitis were either known cases of HIV infection or were immunocompromised. Patients with poor personal and community hygiene, or those who consume contaminated/raw food products, are at risk for ocular parasitic infections.

Unlike studies conducted in other regions of Thailand, this study found panuveitis to be the most common anatomical location. Regarding etiology, infectious causes accounted for 32.8%, with IOTB being the most common specific diagnosis at 6.7%. Compared to other regions, northeastern Thailand showed differences in both clinical presentation and etiology, with a higher prevalence of panuveitis and TB-associated uveitis. Further details regarding studies conducted in other regions of Thailand are listed in Table 4.

It is noteworthy that nearly one-third (31.4%) of the patients in this study have unspecified diagnoses of their intraocular inflammation. The prevalence of this subgroup of patients varies significantly among studies, ranging from as low as 13% in a Thai study [3] to as high as 86.1% in a report from Sweden [30]. Recent research indicates that in the era of PCR, an increasing number of uveitis cases with undetermined etiology are obtaining specific diagnoses through aqueous or vitreous specimen samplings, particularly in cases of infectious etiologies such as viral and toxoplasma infection [31, 32]. Despite a report of low diagnostic utility in one study [33], these positive outcomes have the potential to alter treatment regimens, leading to treatment success [31, 32]. We believe that the availability of PCR and deep sequencing technique will decrease the proportion of patients with idiopathic uveitis.

The most frequent ocular complication in this study was glaucoma, occurring in approximately one-fifth of the patients, a figure comparable to a study from central Thailand [8]. Another Thai study reported cataract as the most common uveitis sequelae [6]. In our clinical practice, cataract grading is subjectively determined by each ophthalmologist and may vary between visits. Therefore, we acknowledge that cataracts may be underestimated as a complication of uveitis.

However, the study has limitations, notably the potential under-recording of CMV retinitis patients as only 21 HIV-infected patients were included. Additionally, cataracts might be underreported as a uveitis complication due to the cross-sectional study design. Long-term follow-up is suggested to accurately determine the incidence of secondary cataracts. Finally, given that our hospital serves as the tertiary uveitis referral center in northeastern Thailand, the majority of our patients were referred from other hospitals. Consequently, the epidemiological data presented in this study may not be directly comparable to findings from smaller-scale healthcare institutions or different geographical regions.

In conclusion, panuveitis predominated among patients in this study. Tuberculosis was the most frequent infectious cause, while VKH was the most common etiology among non-infectious uveitis patients. Glaucoma was a common association with uveitis in this study. Although non-infectious uveitis is observed predominantly in this region, the proportion of infectious uveitis is relatively more common than in developed countries.

## Data Availability

The datasets used and analysed during the current study are available from the corresponding author, WL, on reasonable request.
